# The Awakening of the Hospitals

**Published:** 1919-04-26

**Authors:** 


### The Hospital

The Workers' Newspaper of Administrative Medicine and Institutional Life, Administration, National Insurance and Health.

No. 1716, Vol. LXVI. SATURDAY, APRIL 26, 1919. PRICE TWOPENCE

### THE AWAKENING OF THE HOSPITALS.

The Armistice first brought a feeling of relief to the whole nation, individually and collectively. Ordinary people rejoiced that the horrors of war and its attendant sufferings, its difficulties, trials, and anxieties, with the diminished food supply and inflated prices for all the necessaries of life, had reached their maximum of trouble for the individual, that better days were in sight, and that Peace, with its attendant blessings, might be hoped for with all possible despatch. Institutions, and especially hospitals and those associated with them, had a feeling of relief and thankfulness that the great, continuous, ever-increasing strain upon the individual worker and the resources of the establishment, the continuous call for more beds, more accommodation, more money to keep the work going, must in the near future reverse their incidence and over-pressure give way to gradually diminished claims for personal effort and ever-extending supplies of all kinds. Then, as the months have passed, there has ensued an awakening to the consciousness of the changes which war has produced in regard to hospital establishments, equipment, treatment, and the claims of the civil population, which have had to be put aside to a considerable extent since August 1914. As the war pressure and its never-ending supply of urgent cases, needing relatively the whole resources of the institution to meet the demands of the battlefield, fell off, those responsible for the administration had time gradually to review the position of affairs from the homeland point of view, and the action which would have to be taken to meet the pressing needs of the civil population, and a multitude of middle-class people too, so far as hospital care and treatment are concerned. The Government, too, began to be alive to the requirements which would have to be met by the central authorities, if the fruits of victory were to be provided in due measure for the population of these islands, who had so nobly and whole-heartedly set their faces to win the war, and cause it to result in a better England, a real Merrie England for every resident in Great Britain and throughout the Empire. So, after an interval of five months, things are shaping in the direction of providing, with the aid of the State, in co-operation with the generosity and heartfelt gratitude of those amongst us who have money to give, that plans are being evolved, schemes prepared, and affairs set in motion for the provision of the many matters necessary to the adequate fulfilment of the ideals we have just indicated.

We are proud of our voluntary hospitals, and those of us who know most about them and care most for them are liable, as the days pass, suddenly to awaken to some enormous change for the better which has been quietly worked out and brought into being through the activity and brain-power of some one or more of our great contemporaries in the hospital world. And here we may give a hint to those who realise the privilege of helping our hospitals, taking part in their administration and work, and keeping in touch with the kaleidoscopic changes which have been brought about, especially in recent years, and the splendid thoroughness and efficiency which characterise every detail of the work done in the best administered institutions, quite irrespective of their ancient foundations and the age of the buildings in which the work is being carried on at the present day. Experience has demonstrated that to realise what a severe epidemic may mean in huge blocks of buildings devoted to the care of hundreds or thousands of persons of both sexes, who have continuously been kept under care in great institutions, it is not necessary to hunt up some old and presumably insanitary establishment. The largest and most recent of asylum buildings, planned and built regardless of cost and with infinite care and intelligence, may prove to be the home of a serious epidemic amongst the inmates in the early days of its use as a residence for patients. So, on the other hand, we have in this country, since the institution of King Edward's Hospital Fund for London, types of old hospital buildings which were justly held to require reconstruction or rebuilding when King Edward started his Fund, where the ability, thoroughness, and devotion of a great administrator have produced changes, that have made the old buildings hygienically sound and artistically attractive, and in all particulars efficient for the treatment of acute cases of all descriptions. It is well at the present time to recall these gratifying and indisputable facts. For the greatest danger of the moment is probably the risk that, as a nation, we may be overburdened with demands for new buildings of all kinds in the course of the reconstruction, which the cessation of the war, and the whole-hearted desire of the people to make these islands, in fact, for all classes the Merrie England of our dreams, must inevitably bring. These demands may not only cause the undue expenditure of money but the neglect of opportunities to provide means and accommodation, by the utilisation of existing buildings on the same lines as those which have proved so far successful in reconstructing a few of our greatest and oldest hospitals. This point was made with clearness and force in an article which appeared in these columns on April 12, page 27.

All these considerations, and many others, were brought forcibly to the mind of the writer on Sunday afternoon, when he found himself in a big hospital and invited by the superintendent to go and see the shell of the buildings which were being prepared to provide accommodation for several new departments which the needs of the times and the changes caused by the war have rendered it essential to provide with all possible despatch. These included additional beds for specialisms and children's wards, representing extra accommodation for 104 beds and cots, with a daily occupation of 92, and extra accommodation for nurses by an extension of the Henriette Raphael Nurses' Home; a building containing new departments for massage, Swedish remedial exercises and electricity, with further accommodation for nurses; important extensions and additions to the orthopædic department; a special out-patient department for pulmonary tuberculosis, and a welfare centre for mothers and children in connection with Guy's Hospital. This last has been well thought out, shows great ability in planning and economy of space, with a thorough grasp of the requirements which will have to be met, and promises that this centre, now in course of preparation, will in many respects enjoy unique opportunities and advantages. It is probable that no other welfare centre is so favourably situated in its combination with a midwifery charity, in its close association with a hospital with all the resources of the departments for diseases of children, diseases of the ear, eye, nose, throat, skin, for massage and remedial exercises, and in its relationship with a great medical school, in the laboratories of which problems of physiology, bacteriology, and pathology may be studied and elucidated.

When we had finished our inspection, which involved a good deal of walking, the climbing of scaffolds, and all the many shifts which the inspection of buildings in course of erection include as a matter of course, we were taken through a number of important departments, and were able to examine them with some detail owing to their freedom from patients on Sunday afternoon. We were practical men, keen believers in the enforcement of efficiency in every detail, so that relatively everything, including machinery of all kinds, accommodation for the residents, the arrangements of the casualty department, and the provision for cases of emergency of every kind coming as they are liable to come to a great hospital during any hour of the day or night, were examined. The further we went through this old hospital the more evidence was forthcoming that everywhere efficiency and spotless cleanliness had become possible, by the faithful service and high standard of personal responsibility which every one engaged within its walls had brought to bear upon his or her individual work. Every mechanical device which would save labour, avoid overcrowding and delays in treatment, and ensure the speedy conveyance of patients and staff from any given spot to any part of the hospital buildings, and an infinity of other details, proved how miraculous have been the changes in equipment and in efficiency in the present day in a great and old institution like Guy's Hospital, where the best is good enough for everybody, and less than the best finds no place to rest upon.

We have no space to pursue these thoughts further to-day, except to point the lesson that every hospital worker, and every supporter and believer in these great houses for the sick, should occasionally, at any rate, seek an opportunity to spend an afternoon within their walls, when the workshops are empty, or nearly so, and learn for themselves the magnificence of the administrative efficiency to be found in the best of our hospitals under the voluntary system. This should be the case not only in those places which come constantly and more immediately under the eye of the visitor and the higher officials, but in those portions, too, which are seldom seen, and more often than not may be unrealised and unknown to the majority of visitors, managers, and some of the busiest workers who are tied to one great department. Yet the economical administration, the maximum of the best achievable results, and the triumph of victory over disease and death, are to no small extent dependent upon the presence of the thoroughness exhibited everywhere on the part of those who have to do their work in these establishments far from the public eye. They are indeed all unknown to the majority of the population who every year may find a resting-place and safety within the hospital walls.

